# Basic Symbolic Number Skills, but Not Formal Mathematics Performance, Longitudinally Predict Mathematics Anxiety in the First Years of Primary School

**DOI:** 10.3390/jintelligence11110211

**Published:** 2023-11-01

**Authors:** Patrick A. O’Connor, Kinga Morsanyi, Teresa McCormack

**Affiliations:** 1 School of Psychology, Queen’s University Belfast, Belfast BT9 5AG, UK; t.mccormack@qub.ac.uk; 2 Mathematics Education Centre, Loughborough University, Loughborough LE11 3TU, UK; k.e.morsanyi@lboro.ac.uk

**Keywords:** basic number skills, longitudinal design, math anxiety, mathematics development, order processing

## Abstract

Mathematical anxiety (MA) and mathematics performance typically correlate negatively in studies of adolescents and adults, but not always amongst young children, with some theorists questioning the relevance of MA to mathematics performance in this age group. Evidence is also limited in relation to the developmental origins of MA and whether MA in young children can be linked to their earlier mathematics performance. To address these questions, the current study investigated whether basic and formal mathematics skills around 4 and 5 years of age were predictive of MA around the age of 7–8. Additionally, we also examined the cross-sectional relationships between MA and mathematics performance in 7–8-year-old children. Specifically, children in our study were assessed in their first (T1; aged 4–5), second (T2; aged 5–6), and fourth years of school (T3; aged 7–8). At T1 and T2, children completed measures of basic numerical skills, IQ, and working memory, as well as curriculum-based mathematics tests. At T3, children completed two self-reported MA questionnaires, together with a curriculum-based mathematics test. The results showed that MA could be reliably measured in a sample of 7–8-year-olds and demonstrated the typical negative correlation between MA and mathematical performance (although the strength of this relationship was dependent on the specific content domain). Importantly, although early formal mathematical skills were unrelated to later MA, there was evidence of a longitudinal relationship between basic early symbolic number skills and later MA, supporting the idea that poorer basic numerical skills relate to the development of MA.

## 1. Introduction

A range of everyday tasks necessitate the effective use of numeracy skills, such as managing finances, weighing out ingredients when cooking, or working out how long we have to wait for the next train. The importance of having adequate numeracy skills in everyday life is demonstrated in the finding that individuals with better numerical skills are less likely to be unemployed or suffer from anxiety and depression than their less-numerate peers (National Numeracy 2015). Studies suggest that besides knowledge of relevant facts and procedures, mathematical performance is also dependent on people’s feelings about mathematics. In particular, mathematical anxiety (MA; a feeling of apprehension, unease, or even dread at the prospect of doing mathematics, e.g., Richardson and Suinn 1972) is predictive of mathematical performance. Furthermore, individuals who are anxious about mathematics also tend to show poorer performance in decision-making tasks that require individuals to use their numeracy skills (e.g., Morsanyi et al. 2014; Primi et al. 2018; Rolison et al. 2016), suggesting that the effects of MA are also present in everyday decision-making situations.

Although there is evidence that initial signs of MA are already present in the case of 6-year-old children (e.g., Aarnos and Perkkilä 2012), the early emergence of MA is relatively under-researched. In the current study, we aimed to address some important gaps in this literature by investigating whether the development of MA is linked to impairments in early informal (i.e., skills and concepts that develop before children enter formal schooling, such as counting; Namkung et al. 2019b) or formal mathematical skills (i.e., skills that develop through schooling, such as conceptual and procedural mathematical knowledge, as well as arithmetical skills; Namkung et al. 2019b). Distinguishing between formal and informal mathematics skills in this context is theoretically interesting because a stronger link with formal mathematics skills may suggest that children develop MA as a result of negative feedback on their performance (cf., Ashcraft 2002) or because they experience failure. However, a stronger link with informal mathematics skills would suggest that it is not necessary to experience failure or negative feedback on performance to experience MA. Children with weaker basic mathematics skills may still be able to perform well on tasks, but they may have to invest more time and effort in solving problems and may be less confident in their performance. 

### 1.1. The Emergence of MA

There is no consensus regarding the exact age at which MA first emerges in childhood. Some researchers have suggested that MA develops around fourth grade (9–10 years) once students have encountered more challenging mathematical contents (e.g., Tankersley 1993; Yeo 2005). MA would then continue to increase through the middle school years and reach its peak in the 9th and 10th grades, leveling off during the later high school and college years (Hembree 1990; Vukovic et al. 2013a). In line with this suggestion, some earlier meta-analyses reported that the severity of MA increased with age (Hembree 1990; Ma 1999). Nevertheless, a more recent meta-analysis by Namkung et al. (2019a) found no age-related change in the strength of the relationship between MA and mathematical performance. What is clear is that the link between MA and mathematical performance seems to be well-established by late childhood/adolescence, with the results of meta-analyses suggesting that in studies involving children in grades 4–12 (aged between 9 and 18), the magnitude of the correlation between MA and mathematical performance is between −0.20 and −0.44 (Barroso et al. 2021; Hembree 1990; Ma 1999; Namkung et al. 2019a; Zhang et al. 2019), which is similar to the strength of correlation found in adult samples (e.g., Hembree 1990; Ma 1999). 

However, links between MA and mathematical performance are less clear in younger children (i.e., between the ages of six and eight), with some studies reporting no such link (e.g., Cain-Caston 1993; Cargnelutti et al. 2017b; Dowker et al. 2012; Hill et al. 2016; Krinzinger et al. 2009; Thomas and Dowker 2000), whereas other studies (e.g., Ching 2017; Gunderson et al. 2018; Lauer et al. 2018; Mononen et al. 2022; Primi et al. 2020; Ramirez et al. 2013, 2016; Szczygiel 2020; Tomasetto et al. 2021; Vukovic et al. 2013a, 2013b; Wu et al. 2012, 2014) did find a significant link, even in the first school grades. It is possible that some of these discrepancies reflect the use of instruments that were unsuitable for measuring MA in young children (cf., Primi et al. 2020). More specifically, Primi and her colleagues highlight how papers that report the psychometric properties of appropriate MA scales designed for children have only been published since 2010. Measures of reliability and validity in many of these studies have been less than adequate, and some of these studies are hampered by small sample sizes. Furthermore, there is a lack of studies in which MA has been investigated amongst 6–8-year-olds, with some studies having adapted scales that have been used with adults or are only appropriate for limited age ranges (cf., Primi et al. 2020). Nevertheless, the same review of MA scales for young children (Primi et al. 2020) has shown that there are at least a few scales (Ganley and McGraw 2016; Jameson 2013; Primi et al. 2020; Ramirez et al. 2016; Wood et al. 2012; Wu et al. 2012) that can be reliably used with child samples, with some scales having been successfully used with 6- and 7-year-olds (Primi et al. 2020; Ramirez et al. 2016). In summary, it is not currently clear whether MA shows the same pattern of relationship with mathematical performance in children aged 6–8 years of age as typically observed in older children (aged nine and above) and adolescents.

An additional issue is that, in a developmental context, the direction of any link between MA and mathematics skills needs careful consideration. Although there is currently limited evidence in this regard, a few studies have investigated the longitudinal links and their direction between mathematics performance and MA in young children. In terms of their findings, one study (Cargnelutti et al. 2017a) showed that MA predicted later mathematics performance; two studies found longitudinal links in the opposite direction (Ching et al. 2020; Song et al. 2021); and one study showed reciprocal links (Gunderson et al. 2018). In more detail, Cargnelutti et al. (2017a) showed that MA in grade 2 was related to grade 3 mathematics performance, although this relationship was mediated by grade 2 mathematics performance and grade 3 MA. By contrast, Song et al. (2021) reported that arithmetic fluency in grade 2 predicted the change in MA from grades 2 to 3, but MA did not predict the change in arithmetic fluency across these time points. Furthermore, Ching et al. (2020) conducted an 8-month longitudinal study with 6-year-olds in the first year of elementary school, and they found that quantitative reasoning and number knowledge predicted lower MA at T2. However, earlier MA did not predict later quantitative reasoning and number knowledge. Finally, Gunderson et al. (2018) found evidence for reciprocal relationships between MA and mathematical performance among 7-year-olds (who were first or second graders) in a 6-month longitudinal study. In summary, although there is indication of longitudinal links between MA and mathematical skills in the first school years, the available evidence is limited and contradictory. 

Another important gap in our current knowledge concerns the specific types of mathematical tasks that relate to MA and whether MA relates to all aspects of the mathematics curriculum. Some studies have found evidence that MA is negatively related to certain aspects of formal mathematical performance, such as counting and mathematical concepts (Harari et al. 2013), numerical operations and mathematical reasoning (Wu et al. 2012), and arithmetical problems (Wood et al. 2012). Furthermore, Zhang et al. (2019) reported in their meta-analysis that MA was more strongly related to mathematical problem-solving than computation. Whilst these studies demonstrate that a range of mathematical skills are related to MA, there is little research into whether there are reliable differences between the strength of the relationships between various content areas of the school curriculum and MA and whether all mathematical content areas are related to MA. 

From the perspective of the early development of MA, it could be argued that a more important question is whether there is a link between MA and more basic, potentially foundational, mathematics-related skills that are not typically assessed in school contexts. Maloney et al. (2011) found that MA in adults was related to performance on a symbolic magnitude comparison task (see also Dietrich et al. 2015). On the basis of this finding, Maloney et al. (2011) suggested that MA might stem from impairments in basic mathematical skills that, in turn, might compromise the development of higher-level mathematics abilities. Nevertheless, this was a concurrent correlational study with adults; thus, it does not actually provide evidence regarding causal or developmental links between these skills. Indeed, Song et al. (2021) reported no relationships between MA and number comparison in either 2nd or 3rd graders. Cargnelutti et al. (2017b), also in a cross-sectional study, found that amongst 7–8-year-old children, MA was uncorrelated with several measures of ‘number sense’ (dot comparison, dot estimation, approximate addition), although these measures tend to be related to mathematical performance in children (e.g., Halberda et al. 2008; Lyons et al. 2014; Gilmore et al. 2010). Moreover, Cargnelutti et al. (2017b) also found that MA was unrelated to some non-numerical measures that are typically related to mathematical performance, such as IQ (e.g., Strenze 2007) and both visuospatial and verbal working memory (e.g., Friso-Van den Bos et al. 2013). However, it should be noted that in the same study, there was also no relationship between MA and performance on a formal mathematics test. 

### 1.2. The Aims of the Current Study

The main aim of the current study was to examine MA and its longitudinal predictors in young children. Although there are some existing studies that investigated the longitudinal links between MA and various indicators of mathematical performance in the first grades of primary school, these studies show a mixed picture, with one study suggesting that MA may predict later mathematical skills (Cargnelutti et al. 2017a), whereas other studies showed a link in the opposite direction (Ching et al. 2020; Song et al. 2021), and one study (Gunderson et al. 2018) suggested a reciprocal link. The current study followed a different path from previous studies in that the children in our study were younger at the time of the first measurement point (i.e., they were less than 5 years of age). For this reason, we did not measure MA at the first time point, which did not give us the opportunity to run cross-lagged analyses. Instead, our focus was specifically on exploring whether early difficulties with mathematics-relevant skills, as evidenced by poor performance on tasks assessing either basic mathematical skills or mathematical skills relating to the formal curriculum, are predictive of subsequent MA. Thus, the central aim of our study was to investigate the longitudinal relations between early mathematics-relevant skills and MA. We did this by assessing mathematical and mathematics-relevant skills in the first two years of primary school in Northern Ireland (T1; 4–5 years and T2; 5–6 years) and MA and mathematical performance in year 4 (T3; 7–8 years). 

Year 4 was selected as our target age group for two reasons. First, because MA can be reliably measured by self-report questionnaires from around the age of 7 (see Primi et al. 2020 for a review), we were confident that children in year 4 (who were around the age of 8) would be able to reliably report their levels of MA. Another reason for focusing on year 4 was that the formal mathematical skills of children in Northern Ireland are assessed at the end of their fourth school year to establish whether they have attained the requisite skills to progress to the next key stage of their primary school education (Council for the Curriculum, Examinations and Assessment 2019). This formal testing might provoke some anxiety, which makes the measurement of MA particularly relevant to this age group.

The basic mathematics-related tasks that were selected for the current study have been found to longitudinally predict later mathematical performance. The results of meta-analyses indicate that non-symbolic magnitude skills (as measured by tasks such as non-symbolic addition) are predictive of mathematical performance in children under six (Chen and Li 2014; Fazio et al. 2014) and have been demonstrated to longitudinally predict mathematical performance (Xenidou-Dervou et al. 2017). Symbolic magnitude skills (as measured by number comparison tasks), as well as number line estimation, have also been shown in meta-analytic studies to predict mathematical performance at the age of six and above (Schneider et al. 2017, 2018). Symbolic ordering skills are also predictive of mathematical performance in both cross-sectional (e.g., Attout and Majerus 2018; Lyons and Ansari 2015; Lyons et al. 2014; Sasanguie and Vos 2018; but see Vogel et al. 2015) and longitudinal studies (Liang et al. 2023; Malone et al. 2021; O’Connor et al. 2018). Recent research has demonstrated that non-numerical ordering skills (measured by an everyday ordering questionnaire and a temporal ordering task) measured at age 4–5 are longitudinally predictive of children’s mathematical performance one year later (O’Connor et al. 2018). Performance on temporal ordering and order working memory (WM) tasks have also demonstrated good intra-individual stability as predictors of early formal mathematical skills amongst 4–6-year-olds (O’Connor et al. 2019). Furthermore, order WM skills have been found to longitudinally predict later calculation abilities in children tested between the ages of five and nine (Attout et al. 2014). In spite of all the evidence for the importance of these tasks for early mathematical development, it is not known whether these tasks are also predictive of later MA. Indeed, it is possible that the development of MA is only linked to numerical tasks, as the original conceptualisation of MA as “number anxiety” (Dreger and Aiken 1957) may imply. Furthermore, it is also of interest to investigate whether the longitudinal predictors of mathematical skills and MA are dissociated, which would have implications for the development of interventions designed to reduce anxiety about mathematics and interventions designed to improve mathematical performance. 

Our longitudinal study involved assessing pupils’ performance on a variety of basic cognitive and mathematical tasks at T1 and T2 to examine whether they were predictive of MA at T3. We used a range of numerical and non-numerical skills (including measures of symbolic and non-symbolic magnitude processing, ordering ability, working memory, and verbal and non-verbal intelligence), skills that have been found to be relevant to early mathematical development (O’Connor et al. 2018, 2019). In terms of our outcome measures, we assessed both children’s levels of MA and their formal mathematical performance at the end of year 4, in order to better understand to what extent the early predictors of MA and mathematical performance might overlap. An extreme position regarding MA might state that MA is just another name for being “bad at math” (cf., Beilock and Willingham 2014). Nevertheless, this seems unlikely given the moderate correlations between MA and mathematical performance and the fact that many children with MA show typical or high mathematical performance (cf., Devine et al. 2018). By contrast, if it is the case that early predictors of MA and mathematical performance are largely independent, this would suggest that early intervention efforts should focus on both factors separately.

A further aim of our study was to look more closely at the relationships between MA and a variety of different mathematical skills. MA was first described as “number anxiety” (Dreger and Aiken 1957), and, traditionally, a lot of studies that have investigated the links between MA and mathematical performance have focused on arithmetic skills. The results of meta-analyses (Ma 1999; Zhang et al. 2019) suggest that the relationship between MA and mathematical performance is weaker in studies that index mathematical performance using standardised tests compared to some other outcome measures (such as arithmetic skills), suggesting that MA might not be related (or related equally) to all content areas of the mathematics curriculum. Nevertheless, there is little existing research evidence relating to this question. The Northern Ireland curriculum (Council for the Curriculum, Examinations and Assessment 2019) outlines five key areas in the mathematics curriculum: processes in mathematics (developing approaches to problem-solving, understanding mathematical language, and mathematical reasoning); numbers (understanding numbers, understanding patterns, understanding numerical operations, and understanding money); measures (knowing how to use measurement units, understanding how to tell the time); shape and space (understanding shapes and symmetry); and handling data (collecting, representing, and interpreting data). We used a curriculum-based mathematics test at T3 to assess children’s performance in these content areas and to see whether MA was related to performance in all of these areas.

## 2. Method

### 2.1. Participants

Sixty children^1^ (31 females) were tested during their first (T1) year (mean age = 4 years 11 months, *SD* = 3.82 months), second (T2) year (mean age = 6 years 2 months, *SD* = 3.33 months), and fourth (T3) year of primary school (mean age = 8 years 3 months, *SD* = 3.39 months). Due to the population demographics of Northern Ireland, most children were of Caucasian origin. The children were recruited from four schools in the Belfast and Greater Belfast areas. Information about participants’ socio-economic status, parental education, and children’s IQ can be found in the next section.

### 2.2. Materials

#### 2.2.1. Measures of Socioeconomic Status and Cognitive Ability

##### Northern Ireland Multiple Deprivation Measure

The Northern Ireland Multiple Deprivation Measure (Northern Ireland Statistics and Research Agency 2010), collected at T1, was used as a quantitative indicator of participants’ level of socioeconomic deprivation. Parents were asked to indicate their postcode, which was used to identify the electoral ward in which they lived. A deprivation score is assigned by the Northern Ireland Statistics and Research Agency to each electoral ward in Northern Ireland based on several indices, with higher scores indicative of a greater level of deprivation in that particular area, and can be interpreted as percentiles (e.g., an electoral ward area with a multiple deprivation score of 10 is considered to be less deprived than 90% of all postcode-based electoral ward areas within Northern Ireland). This information is freely available via the Northern Ireland Statistics and Research Agency website. Deprivation scores in the study ranged from 1.85 to 62.91 (Median = 15.79), which was indicative of most participants coming from less deprived areas. One participant did not have a deprivation score assigned to them as their postcode was not provided.

##### Parental Education

At T1, parents were asked to indicate their highest level of qualification. Most parents (68.3%) were at least educated to the undergraduate level. One parent did not indicate their highest level of education, so this score could not be calculated for their child.

##### Verbal and Non-Verbal Intelligence

Intelligence was assessed at T1 and T2 using the Vocabulary and Block Design subtests of the Wechsler Preschool and Primary Scale of Intelligence (WPPSI-III UK; Wechsler 2003) and estimated full-scale IQ scores were calculated using Sattler and Dumont’s (2004) method, with participants demonstrating IQ scores within the normal range at T1 (Mean IQ score = 95.98, *SD* = 14.26) and at T2 (Mean IQ score = 101.72, *SD* = 12.84). In subsequent analyses that included IQ as a covariate, raw scores for both IQ subtasks were entered as separate variables rather than the overall IQ score.

##### Order Working Memory (WM) Task

Based on a task developed by Majerus et al. (2006), this measure assessed participants’ ability to re-create the serial order of a novel sequence of items. Participants were presented with lists of monosyllabic animal names through a set of earphones. After hearing the animal names, participants were given cards that depicted these animals and had to recreate the correct sequence of animals they had heard, from left to right. The length of the item sequences ranged from two to seven, with four trials for each item length. A score of 1 was given for each correctly recreated sequence. Split-half reliability estimates were calculated using the Spearman–Brown formula, which indicated good task reliability at both T1 (*r* = 0.87) and T2 (*r* = 0.95).

##### Daily Event Task

This computerised task was based on a temporal ordering task developed by Friedman (1990) and involved participants verifying the order of familiar daily events. Participants were initially trained on how to order event sequences using two training sequences. The first consisted of a four-card sequence that depicted a child playing on a slide, whereas the second was a six-card sequence that depicted a child picking up and opening a present. During training, participants were asked to recreate the sequences themselves, with feedback given by the experimenter. Once children had demonstrated their ability to re-create each sequence successfully four times in a row, they were then introduced to the experimental set of six daily events (waking up, getting dressed, going to school, eating lunch, eating dinner, and going to bed). Initially, participants were shown the correct order of the six events; the cards were then shuffled, and the participant was asked to re-create that sequence (two trials), with feedback given by the experimenter. In the computerised task, participants were initially presented with a fixation cross in the middle of the screen for 1000 ms. They were then presented with a triad of the daily events, and they had to press a button on the screen to indicate whether the events were in the correct order, from left to right. The triad remained on the screen until the participant made a response. There was a total of 24 trials, with 12 trials presented twice (half of the trials were in the correct order and half were in a mixed order). Participants completed four practice trials (with feedback) before they completed the experimental trials. Each correct response made by a participant was assigned a score of 1. A split-half reliability was calculated (as each trial was presented twice) using the Spearman-Brown method, which was low but acceptable, both at T1 (*r* = 0.59) and at T2 (*r* = 0.68).

#### 2.2.2. Measures of Basic Numerical Skills at the Start of the First School Year

##### Number Ordering^2^

At T1, the task involved children ordering cards depicting the numbers from 1–9. The experimenter initially demonstrated the correct forward order of the digits from 1–9. These cards were then shuffled, and participants were asked to re-create this sequence (in two trials). This process was then repeated for the reversed sequence of numbers from 9–1. Participants were also asked to put the digits in order, from different starting points, in both forward and backward order (four trials for each direction). Accuracy on this task was calculated based on participants’ performance on the four ordering tasks. Participants were given a score of 1 for each correct sequence they were able to create. Cronbach’s alpha for this task indicated a high level of reliability (Cronbach’s alpha = 0.93). A computerised number ordering task was used at T2 (e.g., Lyons and Ansari 2015). Participants initially fixated on a cross presented in the center of the screen for 1000 ms before the presentation of a triad of single digit numbers (displayed in size 200 Arial font). Participants had to press one of two buttons to indicate whether the triad of numbers were in the correct canonical order, from left to right, and this triad stayed on the screen until the participant made a response. The numerical distance (i.e., the numerical distance between the smallest and largest number included in the triad) was manipulated in the task, ranging from 2–7, with eight trials for each numerical distance (48 trials in total). Half of the trials were in canonical order, and half of the trials were in a mixed order. Participants were given a score of 1 for each correct response given. Cronbach’s alpha for task accuracy demonstrated high reliability (Cronbach’s alpha = 0.87).

##### Counting

This was based on a similar task developed by Hannula and Lehtinen (2005). Participants were given two trials in which to count to the highest number they could think of. At T1, if children reached 50, they were stopped. Children at T2 were stopped if they counted to 100. Participants were also asked to count forwards and backwards from different starting points (three trials for each) and were allowed to correct their mistakes. They were stopped once they successfully recited the next three numbers in the sequence, with a score of 1 given for each correct response. At T1, a Cronbach’s alpha reliability estimate demonstrated good reliability for the counting to 50 task (Cronbach’s Alpha = 0.94), and also for the combined forward and backward subtasks (Cronbach’s Alpha = 0.80). Since there were strong, positive correlations between the counting to 50 and both forward; *r* (60) = 0.77, *p* < 0.001) and backward counting; *r* (60) = 0.72, *p* < 0.001), counting performance was calculated by adding together the z-scores for the counting to 50, counting forward, and counting backward tasks, with greater scores representing a greater mastery of counting skills. At T2, the counting to 100 task (Cronbach’s alpha = 0.93) and the combined forward and backward subtasks (Cronbach’s alpha = 0.73) demonstrated good reliability. There were strong, positive correlations between the counting to 100 and both forward; *r* (60) = 0.70, *p* < 0.001) and backward counting; *r* (60) = 0.52, *p* < 0.001); therefore, a counting performance measure for T2 was calculated using the same procedure that was used in T1. 

##### Non-Symbolic Addition

Based on a similar measure by Gilmore et al. (2010), this task involved the approximate addition of two arrays of dots (referred to as the sum array) and the comparison of the sum array to a comparison array. Participants were told that two characters (Paul and Claire) were playing with some marbles and that their task was to work out who had the most marbles each time. In each trial (following a cross appearing on the screen for 1000 ms), Paul’s first array of blue marbles (dots) appeared, and then moved down the screen to be hidden behind an occluder. Paul’s second array of marbles appeared, and again moved down the screen behind the occluder. Then, Claire’s set of red marbles (dots) appeared and moved down to the bottom of the screen. Participants were then presented with pictures of the characters and had to press the picture of the character who they thought had the most marbles. 

These marbles were represented in the study as coloured dots which varied in size (the large dots were 20 pixels wide, whereas the smaller dots were 10 pixels wide). The numerical ratio between the sum and comparison arrays was either 1:2, 3:5, or 2:3, with eight trials for each ratio (24 trials in total). Participants were initially given four practice trials (with feedback provided). The number of dots in each of the arrays ranged from 6 (to reduce the likelihood of children simply subitizing the array) to 45. The key perceptual characteristics of the arrays were dot size (large vs. small), array size (large overall array vs. small overall array), and density (dense vs. spread), which were systematically manipulated so that they were correlated with numerosity on half of the trials (congruent trials) and uncorrelated with numerosity on half of the trials (incongruent trials), to reduce the likelihood that children relied on perceptual cues in order to solve the task. Also, the task was designed so that participants could not perform above chance if their responses were based on comparing the second of the two sum arrays with the comparison array. The comparison array was at least 1.5 times greater in number than the second sum array on each trial. The numerosity of the comparison array was larger than the overall sum array on half of the trials, whereas for the other half of the trials, the opposite was true. A score of 1 was given for each correct response. The Cronbach’s alpha reliability estimate indicated that reliability for this task was low but acceptable at both T1 (Cronbach’s alpha = 0.53) and T2 (Cronbach’s alpha = 0.63). Furthermore, a series of one-sample t tests provided confirmation of children’s performance being above chance at each ratio at both time points. 

##### Number Comparison

This was a computerised task in which participants were presented with a target number (1, 2, 3, 4, 6, 7, 8, or 9) and had to judge whether this target was larger or smaller in magnitude than five by pressing either a large square (if the digit was larger in magnitude than five) or a small square (if the digit was smaller in magnitude than five) on the screen. Participants fixated on a cross for 1000 ms before the target number was shown on the screen, with each target number being presented five times (40 trials in total), with a score of 1 given for each correct response. Participants completed four practice trials initially (with feedback). A Cronbach’s alpha reliability estimate indicated high reliability at T1 (Cronbach’s alpha = 0.88), and an acceptable reliability estimate at T2 (Cronbach’s alpha = 0.65).

##### Number Line Task

This task was based on a similar task (Aagten-Murphy et al. 2015), in which participants were told that they had to help Postman Pat deliver presents to several houses on different streets. Participants were shown a target number and they had to indicate the position of the number on the number line by using their finger to press the relevant position on the screen. Both 1–10 and a 1–20 number lines were used, and both lines were of the same length (1000 pixels). Participants completed two practice trials and six experimental trials for each scale. Participants’ error on each trial (the difference in pixels between the correct and estimated position of the number) was averaged across both scales to provide an overall measure of performance. A Cronbach’s alpha reliability estimate indicated that task performance demonstrated an adequate level of reliability at T1 (Cronbach’s alpha = 0.71) and at T2 (Cronbach’s alpha = 0.70).

##### Mathematics Performance

At the end of participants’ first year of primary school, their level of mathematical performance was measured via a composite 28-item test, comprising arithmetical problems (addition and subtraction) from the calculation subtest of the Woodcock-Johnson III tests of achievement (Woodcock et al. 2001) and questions involving counting items, selecting the next number after a given number in the counting list, as well as selecting the larger number from a choice of two, taken from the Test of Early Mathematics Ability (TEMA-3; Ginsburg and Baroody 2003). Participants completed the assessment in small groups (3–6), in which the experimenter read each of the questions out to the participants, who were instructed to write down their answers. 

Mathematical performance at T2 and T3 was measured using the curriculum-based Mathematics Assessment for Learning and Teaching (MaLT; Williams 2005). The MaLT assessment has been standardised using data from over 12,500 pupils and is purported to be used for monitoring children’s progress in mathematics as well as for individual diagnostic profiling (Hodder Education n.d.). The mathematical performance assessment at T2 (MaLT 5) consisted of 30 questions, whilst the T3 version (MaLT 7) consisted of 45 questions. These tests assessed relevant, age-appropriate areas of the mathematical curriculum. The T2 measure assessed: counting and understanding numbers, knowing, and using number facts, calculating, and measuring. The T3 measure assessed the same areas as the T2 measure, with the addition of understanding shape and handling data. As was the case in T1, participants completed the T2 assessment in small groups (3–6), in which the experimenter read each of the questions out to the participants, who were instructed to write down their answers. The test took 45 minutes for each session. Participants’ raw scores on these mathematical performance measures were used in subsequent analyses. Participants completed the T3 assessment in groups of 7–15, and they read the questions themselves and wrote down their answers. Cronbach’s alpha reliability estimates demonstrated high reliability for the mathematical assessments measured at each time point (T1 Cronbach’s alpha = 0.91; T2 Cronbach’s alpha = 0.83; T3 Cronbach’s alpha = 0.87).

##### Mathematical Anxiety

Two scales were used to measure MA at T3. The first scale was the Revised Child Math Anxiety Questionnaire (CMAQ-R; Ramirez et al. 2016), a 16-item scale that assesses MA in several school-based scenarios. Participants indicated their level of nervousness for each item by pointing to a five-point Likert scale involving smiley faces. In the current study, the scale demonstrated good internal consistency (Cronbach’s alpha = 0.88). The second scale was the Early Elementary School Abbreviated Math Anxiety Scale (EES-AMAS; Primi et al. 2020), containing nine items concerning mathematical situations in a school setting. Participants indicated their level of anxiety about each situation using a five-point Likert scale with squares that increased in the amount of space that was filled up within them, which represented increasing anxiety. The experimenter read all of the items aloud to the participants and administered these scales to the children individually at T3 prior to conducting the group mathematics achievement session. The EES-AMAS scale also demonstrated good internal consistency (Cronbach’s alpha = 0.89). A composite score was calculated by summing the z scores for both scales because there was a strong, positive correlation between anxiety ratings on the CMAQ-R and EES-MAS scales (*r* (60) = 0.81, *p* < 0.001). This composite MA measure was used for all subsequent analyses. 

### 2.3. Procedure

The study received ethical approval from the first author’s university departmental ethics committee. The testing at T1 and T2 was split into two separate sessions, which were identical across time points. Participants completed the Number Ordering task, Number Comparison task, Order WM, and the Non-Symbolic Addition task in the first session. In the second session, participants completed the Daily Events task, the intelligence measures, the Counting task, and the Number Line task. The order of the tasks was the same for all participants and was the same at both T1 and T2. Computerised measures were designed using E-Prime Version 2.0 and presented on a touchscreen monitor that was connected to a Dell laptop. At the end of the school year, at each time point, participants’ math performance was assessed. At T3, participants completed the CMAQ-R, followed by the EES-AMAS and the formal math performance measure at the end-of-school year measurement point.

### 2.4. Power Analysis

A power calculation was carried out using G*Power 3.1.9.7 software. A bivariate correlation analysis was specified, assuming the ability to detect a medium effect size of f = 0.3 (Cohen 1988), which corresponds to the average size of correlation found between MA and mathematics performance in meta-analyses (see Barroso et al. 2021; Hembree 1990; Ma 1999; Namkung et al. 2019a; Zhang et al. 2019). With an α = 0.05 and a power (1 − β) = 0.80, the power analysis yielded a total minimum sample size of N = 67^3^.

## 3. Results

### 3.1. Concurrent MA and Math Performance at T3

Descriptive statistics for MA and mathematical performance at the end of year 4 of primary school (T3) are shown in Table 1, alongside descriptive statistics for mathematical performance at T1 and T2. Performance on the curriculum-based mathematics tests at the end of years 1, 2, and 4 showed a good spread, and there was no sign of floor or ceiling effects. The standardised scores on the mathematical performance measure at T3 also indicated that children’s scores were within the normal range. 

We first looked at the nature of the relationship between MA and mathematical performance (both overall performance and performance on each content area within the assessment) at T3. The purpose of this analysis was to investigate the concurrent relationships between MA and mathematical performance at the end of children’s fourth year of primary school. Furthermore, the analyses also afforded a more in-depth assessment of the type of mathematical content that may be negatively associated with higher levels of MA. As shown in Table 2, Pearson’s correlations indicated that MA was negatively and significantly correlated with overall T3 mathematical performance and some of the mathematical content areas. The Bayesian analysis demonstrated moderate evidence for a correlation between overall mathematical performance and MA; strong evidence for a correlation with counting and understanding numbers; and moderate evidence for correlations with knowing and using number facts and calculating. The Bayesian analysis also indicated that there was moderate evidence for no relationship MA in the case of understanding shape, measuring, and handling data. 

### 3.2. Longitudinal Relations between T1 and T2 Basic Skills and T3 Math Performance

We next examined whether there was evidence of longitudinal correlations between the measures of basic cognitive and mathematics-relevant skills taken at T1 and T2 and mathematical performance measured at T3 (Table 3). Examining these relationships is interesting both from a theoretical and practical perspective, in terms of identifying the early predictors of emerging formal mathematics skills and potential targets for interventions. Mathematical performance at T3 was significantly and positively correlated with several measures, as shown in Table 3. The directional Bayesian analysis (which predicted that the variables would be correlated positively) demonstrated extreme evidence for a correlation between mathematical performance and number ordering at T2, non-symbolic addition at T2, and counting at T1. The analysis also demonstrated very strong evidence of a correlation between mathematical performance and daily event ordering at both time points and number line estimation at T1. There was also moderate evidence of a correlation between mathematical performance and order WM at both time points, as well as with number comparison at T2. The analysis also demonstrated anecdotal evidence for no correlation with mathematical performance for the parental ordering questionnaire at T1, number ordering at T1, non-symbolic addition at T1, number comparison at T1, counting at T2, and number line estimation at T2. 

Partial correlations, controlling for parental education, verbal and non-verbal IQ, and socioeconomic status at T1, indicated that mathematical performance remained significantly and positively correlated with counting at T1 (*r* (51) = 0.41, *p* = 0.002). number line estimation at T1 (*r* (51) = 0.40, *p* = 0.003), order WM at T2 (*r* (51) = 0.37, *p* = 0.007), and number ordering at T2 (*r* (51) = 0.35, *p* = 0.010). Overall, the longitudinal correlations indicated that most basic early predictors of mathematical performance (including both symbolic and non-symbolic number skills, as well as non-numerical ordering ability) were significantly related to mathematical performance over 2 or 3 years later, and some of these variables were still predictive of later mathematical performance when the effects of children’s intelligence and socioeconomic background were controlled.

### 3.3. Longitudinal Relations between T1 and T2 Basic Skills and MA at T3

The reasoning for our next analysis was that evidence of longitudinal correlations between early mathematical performance and later MA may suggest that poor early mathematical performance (or experience of negative feedback on early mathematics performance) might contribute to the later development of negative feelings about mathematics. Furthermore, correlations between basic cognitive and mathematics-relevant skills and MA may suggest specific skills that could be targets for intervention. We also wanted to investigate whether the early correlates of MA and mathematics performance dissociate, which is another theoretically interesting question.

In contrast with the above findings relating to the predictors of T3 mathematical performance, MA was only significantly (negatively) correlated with number comparison at T1 and number ordering at T2. The directional Bayesian analysis (which predicted that the variables would be correlated negatively) demonstrated strong evidence for a correlation between mathematical performance and number ordering at T2 and with number comparison at T1. The Bayesian analysis also demonstrated anecdotal evidence for no correlation with MA for daily event ordering at T1, counting at both time points, and non-symbolic addition at both time points. There was moderate evidence for no correlation between MA and daily event ordering at T2, OPQ and order WM at both time points, and the number line at T2. Finally, there was also strong evidence for no correlation between MA and number line estimation at T1 and number ordering at T1. 

After controlling for the covariates, the correlation between MA at T3 and number comparison at T1 and between MA at T3 and number ordering at T2 remained significant. Additionally, there was now a marginally significant negative correlation between MA and counting at T1 (*r* (52) = −0.26, *p* = 0.061) and a significant negative correlation between MA and counting at T2 (*r* (52) = −0.32, *p* = 0.017). Overall, these results suggested that among the early, basic predictors of mathematical performance, only symbolic number skills were longitudinally related to MA. Nevertheless, these relationships were robust.

### 3.4. Longitudinal Relations between Formal Mathematical Skills at Different Time Points and between Formal Mathematics Skills and MA

We investigated the stability of formal mathematical performance over time by assessing the longitudinal links between the mathematical measures taken at the end of the school year. This question is of interest, as children may start their formal schooling with different levels of mathematics knowledge due to differential access to both formal and informal mathematics activities at home and in early years settings. Nevertheless, this early knowledge gap may reduce in the first school years. Additionally, we were also interested in whether early formal mathematics skills predict later MA and how the strength of this relationship compares to the correlation between early informal mathematics skills and MA. Formal mathematical skills at T3 were strongly correlated with formal mathematical skills at T1 and T2, and the Bayesian analyses also suggested extreme evidence in favour of the alternative hypothesis for both. After controlling for the covariates, formal mathematical skills at T1 and T3 (*r* (53) = 0.44, *p* = 0.001), and at T2 and T3 (*r* (53) = 0.45, *p* < 0.001) were still robustly related. By contrast, early formal mathematics skills at T1 and T2 were not significantly related to MA at T3. The Bayesian analyses indicated moderate evidence for no relationship between formal mathematical performance at T1 and MA at T3, and anecdotal evidence for no relationship between formal mathematical skills at T2 and MA at T3.

### 3.5. Indirect Effects of Early Formal Math Skills on T3 MA

**Figure 1:**
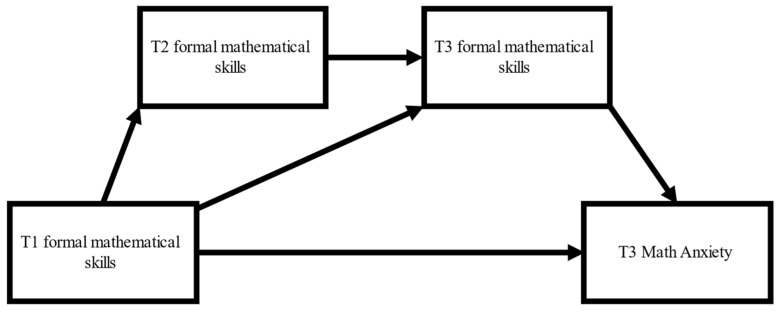
Theoretical model demonstrating the potential mediational effects of mathematical performance at T2 and T3 on the relationship between T1 mathematical performance and MA measured at T3.

In our final analyses, we tested the possibility that even though early formal mathematical skills at T1 and T2 were not significantly related to MA at T3, they may have impacted T3 MA indirectly, via T3 formal mathematical skills. To test for these possibilities, we first conducted a serial mediation analysis using Preacher and Hayes’s (2008) INDIRECT regression procedure with 10,000 bootstrapped samples to estimate the 95% confidence intervals (CIs) for the following indirect pathways: the pathway between T1 formal mathematical scores and T3 MA via T2 formal mathematical skills (indirect pathway 1), T3 formal mathematical skills (indirect pathway 2), and T2 and T3 formal mathematical skills in serial (indirect pathway 3). Figure 1. provides a visual presentation of the possible mediational pathways. The INDIRECT procedure makes it possible to test the potential effects of several mediators, as well as potential serial mediation effects, in a single analysis without the need to conduct separate analyses to statistically compare the adequacy of competing models. 

In line with the correlational analyses, the total effect of T1 formal mathematical skills on MA was not significant (*p* = 0.907). Indirect pathways 1 and 2 also showed non-significant effects. However, the serial mediation effect (i.e., indirect pathway 3) was significant (b = −0.57; 95% CI: −1.31 to −0.03). The direct effect of T1 mathematical skills on T3 MA was approaching significance (*p* = 0.062). Nevertheless, the serial mediation effect became non-significant after controlling for the effects of T1 IQ and SES.

**Figure 2:**
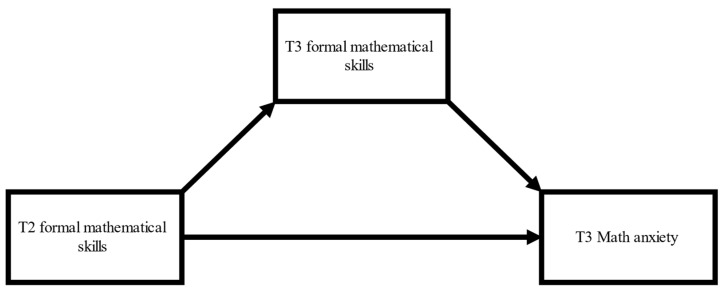
Theoretical model demonstrating the potential mediational effect of mathematical performance at T3 on the relationship between T2 mathematical performance and math anxiety measured at T3.

We also conducted a mediation analysis (again, using the INDIRECT procedure) to investigate the potential indirect link between T2 formal mathematical skills and T3 MA via T3 formal mathematical skills (Figure 2). In line with the correlational analyses, the total effect of T2 formal mathematical skills on T3 MA was not significant (*p* = 0.149). However, the model showed that T3 formal mathematical skills were a significant mediator of the link between T2 mathematical skills and T3 MA (b = −0.82; 95% CI: −1.70 to −0.06). The direct effect of T2 mathematical skills on T3 MA was non-significant (*p* = 0.926). Nevertheless, the mediation effect became non-significant after controlling the effects of T2 IQ and SES.

## 4. Discussion

In the current study, we were interested in several research questions. First of all, we wanted to investigate the longitudinal relations between T1 and T2 basic and formal mathematics-related skills and T3 MA. We were also interested in whether the early longitudinal predictors of MA and formal mathematics skills dissociate, suggesting different developmental pathways. We tested children during their first and second years of primary school and at the end of their fourth year of school. This is an important time in the Northern Ireland school system because children have to complete a formal mathematical skills assessment, designed to establish whether they have attained the requisite skills to progress to the next stage of their primary school education (Council for the Curriculum, Examinations and Assessment 2019). 

In the study, both MA scales showed good reliability and were strongly correlated, confirming that it is possible to reliably assess MA in 7–8-year-olds using self-report measures (see also Primi et al. 2020 for a review). Additionally, our results demonstrated evidence of a moderate, negative correlation between MA and mathematical performance at 7–8 years of age, which is similar to the effect sizes reported in meta-analyses that investigated the link between MA and mathematical performance in adolescents and adults (e.g., Barroso et al. 2021; Ma 1999; Namkung et al. 2019a; Zhang et al. 2019). Nevertheless, not all content areas of the mathematics curriculum were linked to MA. There was evidence for a negative relationship between MA and counting and understanding number (e.g., counting money), knowing and using number facts (e.g., solving arithmetic problems), and calculating (e.g., writing an equation), whereas the content areas of understanding shape (e.g., finding hexagons among a set of shapes), measuring (e.g., reading off the weight of an object from a mechanical scale), and handling data (e.g., interpreting graphs) were unrelated to MA. These results extend previous findings that showed that MA may not be equally strongly linked to all aspects of mathematics performance (e.g., Zhang et al. 2019). 

Although these findings are novel and would need further investigation in future studies, a potential interpretation is that MA is not necessarily triggered by all mathematics contents but is specifically linked to numbers, as suggested by the original concept of “number anxiety” (cf., Dreger and Aiken 1957). Indeed, in the Northern Ireland mathematics curriculum, the three contents that were negatively related to MA would fall under the general ‘number’ moniker in terms of the skills that children at this age must be able to acquire (Council for the Curriculum, Examinations and Assessment 2020). According to the curriculum, these three mathematical contents that children were anxious about concern skills such as counting, understanding how to use the four different types of operations, and mental addition and subtraction of double-digit numbers. Given that these skills are heavily dependent upon knowledge and understanding of the symbolic number system, this suggests that the extent to which children at age 7–8 have mastered the number system may determine their level of anxiety about mathematics. 

This suggestion is also supported by the findings regarding the longitudinal relations between early mathematics-related skills and later mathematical performance and MA. It is notable that formal mathematical performance in year 4 was not only related to formal mathematical performance in years 1 and 2, but also to more basic mathematics-related cognitive skills, including order memory and non-numerical ordering ability, counting, number comparison, number ordering, number line estimation, and non-symbolic addition. By contrast, when the effects of children’s verbal and non-verbal intelligence and their SES were controlled, MA was only significantly predicted by counting at T2, number comparison (at T1), and number ordering performance (at T2). These findings suggest that from the various robust early predictors of mathematical performance, only the basic tasks relating to symbolic number knowledge (and children’s understanding of the relations between symbolic numbers) were predictive of later MA. 

The finding that early symbolic skills predicted later MA is somewhat in agreement with the findings of Maloney et al. (2011), who found that adults who were highly anxious about mathematics performed poorer on numerical comparison compared to their less anxious peers (see also Dietrich et al. 2015 and Núñez-Peña and Suarez-Pellicioni 2014 for related findings). Maloney et al. (2011) reasoned that this was indicative of MA possibly developing from deficits in basic symbolic skills. In the current study, performance on two basic symbolic skills (number ordering and comparison) was predictive of later MA. Both of these skills have been found to emerge as important predictors of mathematical development during the first years of schooling (Sasanguie and Vos 2018). Further investigation may be carried out into the efficacy of training these skills in order to attenuate MA. 

Taken together, these results make some interesting contributions to our understanding of the early development of MA. First, the fact that early formal mathematics skills are not predictive of later MA suggests that it is unlikely that MA first arises as a result of negative experiences with formal mathematical tasks. In other words, children’s experiences with mathematics at school (and the feedback they receive on their performance) might not be the main sources of MA at this early stage. At the same time, our results regarding the relations between MA and both number comparison and number ordering performance lend support to the notion that MA might stem from impairments in very basic mathematical skills (e.g., Dietrich et al. 2015; Maloney et al. 2011) that might subsequently compromise the development of higher-level mathematics abilities. The close relationship between number comparison and number ordering skills is well documented in the literature (Morsanyi et al. 2017, 2020; Sasanguie et al. 2017; Sasanguie and Vos 2018), as well as the importance of these tasks for the development of later mathematics skills (e.g., Brankaer et al. 2017; Lyons et al. 2014; Nosworthy et al. 2013). It is also notable that out of all the basic early predictors that we considered in this study, only number ordering performance at T2 was significantly correlated with both MA and mathematical performance at T3, further confirming that this task taps into an important foundational skill for later mathematical performance. Indeed, although this task measures a very simple and basic skill, in the case of adults, it is strongly related to both complex arithmetic skills (Lyons and Beilock 2011) and mathematical reasoning performance (Morsanyi et al. 2017). 

The fact that there was very little overlap between the early longitudinal predictors of MA and mathematical performance suggests that mathematical difficulties and MA have different origins. This is in line with the findings of Devine et al. (2018), who reported that among primary and secondary school students, 77% of pupils with high MA showed average or high mathematics performance. In other words, although problems with some basic numerical processes might contribute to the development of MA, much of the variance in MA is explained by other factors.

An interesting finding concerned the role of the Approximate Number System (ANS), which is proposed to be an important precursor to the development of symbolic number knowledge (e.g., Piazza et al. 2010). Although non-symbolic addition correlated with later mathematical performance, this correlation became non-significant after controlling for the effects of the covariates. Moreover, this measure was unrelated to MA. This result is consistent with Barroso et al. (2021), who also found in their meta-analysis that measures tapping into the ANS were unrelated to MA. This suggests that although these skills may be important to early numerical development, they are not predictive of the development of later negative feelings about mathematics.

### 4.1. Limitations and Future Directions

A limitation of the current study is that we did not include a measure of MA at each time point, so it is difficult to ascertain exactly how the relationship between MA and mathematical performance develops over the first few years of primary school. We were also not able to establish whether early basic numerical skills contributed to growth in MA over the first year of primary school. Nevertheless, so far, the youngest age when studies have been able to reliably measure MA using self-report scales was 6–7 years (e.g., Primi et al. 2020; Ramirez et al. 2013; 2016; Tomasetto et al. 2021; Vukovic et al. 2013a; Wu et al. 2012; Wu et al. 2014), and it is questionable whether this approach could work with 4–5 year-old children. An alternative approach could be to ask parents or teachers for anxiety ratings in the case of very young children (see Cargnelutti et al. 2017a).

A related potential criticism is that we did not collect other measures of anxiety in addition to MA. Research suggests that there is a link between MA and other forms of anxiety, including test anxiety (e.g., Ashcraft et al. 1998; Devine et al. 2012; Dowker et al. 2016; Hembree 1990; Morsanyi et al. 2014), general anxiety (e.g., Ashcraft and Ridley 2005; Cargnelutti et al. 2017a; Dowker et al. 2016; Hembree 1990; Hill et al. 2016; Wang et al. 2014), and health anxiety (Levy et al. 2014; Rolison et al. 2020). In the case of adolescents and adults, there is good evidence that the link between MA and mathematical performance remains significant after controlling for the effects of other types of anxiety (see Hembree 1990 for a meta-analysis). Nevertheless, in younger children (i.e., aged six to eight years old), general anxiety could be a particularly relevant factor in relation to mathematical performance (cf., Cargnelutti et al. 2017a). Future studies could investigate further whether MA relates to mathematical skills in early elementary school children when the effects of general anxiety are taken into account. 

Attenuating the development of MA should be of utmost interest to educators, as it could interfere with mathematics learning starting from the first years of school (Tomasetto et al. 2021), leading to cumulative gaps. Future research could also involve controlled experimental studies to investigate whether specific interventions can attenuate MA. In one study (Passolunghi et al. 2020), the authors compared three training methods to attenuate math anxiety (one strategy focused on emotional training to reduce MA; another focused on improving mathematical abilities and mathematical strategy use via activities involving multiplication and division; whilst another was a control condition in which children created and drew comic strip stories). Nine-year-old children were assigned to one of these three conditions. The authors found that MA training reduced MA but did not improve mathematical performance, whereas practicing useful mathematical strategies led not only to improved mathematical skills but also to a reduction in MA. Further studies could investigate whether such training would be effective in attenuating the development of MA among children of the same age as in the current study.

Order WM performance at both T1 and T2 moderately correlated with mathematical performance but was unrelated to MA. The link between mathematics performance and WM is unsurprising, as there is a plethora of evidence that almost all forms of mathematics rely on WM resources, with the exception of some highly practiced, automatised processes that are based on simple memory retrieval (see Friso-Van den Bos et al. 2013 for a review and meta-analysis). This relationship might be particularly strong in the first grades of primary school (Lee and Bull 2016). Whilst it is also well-established that MA compromises the functioning of WM (see Ashcraft and Krause 2007 for a review), there is little evidence that WM capacity, *per se*, would be related to MA. Thus, our findings are in line with the existing literature in this respect (Finell et al. 2022; Friso-Van den Bos et al. 2013; Peng et al. 2016). Nevertheless, future research could investigate further the nature of the relationship between WM, MA, and numerical skills in younger children. 

The current study demonstrated that MA can be reliably measured in 7–8-year-old children. However, a gap in the literature exists concerning whether it is possible for MA to develop at a younger age. Whilst previous research has demonstrated that MA can be measured in children as young as six years old (Primi et al. 2020; Ramirez et al. 2016), a suggestion for future research could be to investigate whether MA is linked to mathematical performance in children under the age of six, either through the development of age-appropriate scales or via parent and/or teacher ratings of children’s MA. 

Although we did not consider the effects of other types of anxiety in addition to MA, a strength of our design was to control for the potential effects of children’s socioeconomic status, parental education, and verbal and non-verbal intelligence. Controlling the effect of contextual variables and children’s ability levels allows for drawing stronger conclusions regarding the specificity of the links between MA and both basic and more complex mathematics skills. A final limitation concerns the relatively small sample size, due to the unavailability of a large proportion of our original sample at T3. Nevertheless, the children included in our final sample did not differ significantly from the original sample in their demographic characteristics or ability levels, and our key findings were shown to be statistically robust. This was evident from both the Bayesian analyses and the fact that our results (with the exception of the link between formal mathematical abilities and MA at T3) remained significant after controlling for the effects of various covariates.

### 4.2. Conclusions

In conclusion, the current study demonstrated that MA can be reliably measured using self-report scales among 7–8 year-olds. Whereas we did not find strong evidence for a relationship between MA and earlier formal mathematical skills at this age, we found evidence that early basic symbolic number skills longitudinally predicted later MA. There is very little research that investigates the potential longitudinal relations between MA and mathematical performance amongst early elementary school children. Nevertheless, three existing studies (Ching et al. 2020; Gunderson et al. 2018; Song et al. 2021) with children in their early school grades reported similar longitudinal links to our findings, with early symbolic number skills being linked to growth in MA. Interestingly, out of these studies, only Gunderson et al. (2018) found evidence for reciprocal relationships between MA and mathematical performance. The current study makes novel contributions by investigating these longitudinal relations starting from an earlier age and including measures of formal mathematical skills as well as a range of basic mathematical and mathematics-related skills, while also carefully controlling the effects of important covariates, such as socio-economic status and children’s IQ. Furthermore, while the small sample size and lack of additional measures of anxiety were drawbacks of the current study, we were also able to demonstrate that MA was specifically related to those aspects of the mathematics curriculum that involve the processing of numerical information, which is a novel finding in the literature. In summary, the current study is the first to demonstrate that impairments in early symbolic number skills (but not in formal mathematics skills) are linked to the emergence of MA in the first years of elementary school. Moreover, we demonstrated the content-specificity of both the longitudinal and cross-sectional relations between mathematical skills and MA. These results may be taken as indications that training in early number skills could potentially be useful in reducing the likelihood of children becoming anxious about mathematics in their later years of primary school (see Passolunghi et al. 2020 for related findings). Additionally, our findings are in apparent contrast with earlier claims that MA only emerges in later school years, and in particular, when children encounter more complex and challenging mathematical contents (e.g., Tankersley 1993; Yeo 2005). Future studies could also investigate whether MA relates to mathematical skills in early elementary school children after controlling for the effects of general anxiety.

## Figures and Tables

**Table 1:** Descriptive statistics for the mathematical anxiety and mathematical performance measures and *p*-values for Kolmogorov-Smirnov normality tests.

	Mean (SD)	Range	*D*
Parental education (Median)	5	0–6	<0.001
Deprivation (Median)	10.09	1.85–62.91	<0.001
Vocabulary (T1)	15.93 (7.03)	7–32	0.030
Block design (T1)	24.40 (3.37)	16–30	0.011
OPQ (T1)	44.12 (7.18)	26–56	0.200
Order WM (T1)	9.93 (4.36)	1–16	<0.001
Order WM (T2)	11.23 (4.34)	1–19	<0.001
Daily events (T1)	0.66 (.12)	0.46–0.96	<0.001
Daily events (T2)	0.77 (.12)	0.46–1	0.003
Number ordering (T1)	0.85 (.28)	0–1	<0.001
Number ordering (T2)	0.76 (.20)	0.38–1	<0.001
Counting (T1)	0.20 (2.72)	−8.21–3	<0.001
Counting (T2)	0.32 (1.83)	−9.28–1	<0.001
Non-symbolic addition (T1)	0.56 (0.11)	0.30–0.88	0.002
Non-symbolic addition (T2)	0.66 (0.14)	0.33–0.96	0.178
Number comparison (T1)	0.71 (0.19)	0.40–1	0.008
Number comparison (T2)	0.97 (0.04)	0.75–1	<0.001
Number line estimation (T1)	199.36 (78.52)	64–453	0.023
Number line estimation (T2)	120.24 (42.82)	42.222	0.200
CMAQ-R	34.30 (13.30)	16–70	0.200
EES-AMAS	19.53 (9.18)	9–39	<0.001
Math Performance at T1	23.24 (4.88)	1–28	<0.001
Math Performance at T2 (raw score)	21.74 (4.71)	7–29	0.001
Math Performance at T3 (raw score)	27.43 (7.15)	13–40	0.200
Math Performance at T3 (standardised score)	106.10 (11.49)	86–130	0.200

Task Abbreviation: CMAQ-R: Revised Child Math Anxiety Questionnaire. EES-AMAS: The Early Elementary School Abbreviated Math Anxiety Scale. OPQ: Order-Processing Questionnaire. WM: Working Memory.

**Table 2:** Pearson’s and Bayesian correlations between math anxiety and both overall math performance and performance on the different domains of the math performance assessment at T3.

	Math Anxiety		
	*r*	BF_10_	BF_01_
Formal math performance (Raw)	−0.31 *	5.380	0.186
Counting and understanding number	−0.35 **	12.434	0.080
Knowing and using number facts	−0.30 *	4.418	0.226
Calculating	−0.28 *	3.210	0.312
Understanding shape	−0.07	0.263	3.807
Measuring	−0.07	0.267	3.748
Handling data	−0.08	0.289	3.464

* *p* < 0.05, ** *p* < 0.01.

**Table 3:** Zero-order correlations (controlling for IQ and SES) between math anxiety and performance at T3 and the measures at T1 and T2.

	Formal Math Performance (T3)			Math Anxiety (T3)		
	*r*	BF_10_	BF_01_	*r*	BF_10_	BF_01_
OPQ (T1)	0.21	1.03	0.97	0.01	0.15	6.55
Order WM (T1)	0.28 *	3.23	0.31	0.07	0.11	8.89
Order WM (T2)	0.30 *	4.62	0.22	0.08	0.11	9.41
Daily events (T1)	0.41 **	62.23	0.02	−0.12	0.40	2.47
Daily events (T2)	0.39 **	30.95	0.03	−0.01	0.17	6.01
Number ordering (T1)	0.25	1.73	0.58	0.13	0.09	11.45
Number ordering (T2)	0.49 ***	663.18	<0.01	−0.35 **	13.56	0.07
Counting (T1)	0.44 ***	143.21	<0.01	−0.24	1.67	0.60
Counting (T2)	0.20	0.95	1.05	−0.17	0.63	1.60
Non-symbolic addition (T1)	0.23	1.39	0.72	−0.22	1.26	0.79
Non-symbolic addition (T2)	0.45 ***	197.52	<0.01	−0.22	1.15	0.87
Number comparison (T1)	0.14	0.49	2.05	−0.37 **	20.90	0.05
Number comparison (T2)	0.32 *	6.25	0.16	−0.04	0.21	4.87
Number line estimation (T1)	0.35 **	12.60	0.08	0.12	0.38	2.61
Number line estimation (T2)	0.16	0.60	1.67	0.07	0.26	3.83
Formal math performance (T1)	0.47 **	340.31	<0.001	0.02	0.15	6.79
Formal math performance (T2)	0.58 **	28,939.12	<0.001	−0.19	0.82	1.22

* *p* < 0.05, ** *p* < 0.01, *** *p* < 0.001. Task Abbreviation: OPQ: Order-Processing Questionnaire. WM: Working memory.

## Data Availability

The final data file is available at https://doi.org/10.17605/OSF.IO/JFNQC.

## Notes

1 The original sample consisted of ninety children at T1. At T2, two of the children moved schools, and one further participant was withdrawn from the study. At T3, a further 27 children did not take part in the study. Three of these children moved to another school. The remaining 24 children did not take part because their parent(s) did not give consent for their child to take part. The children who did not take part at T3 were not all from the same class (children in the study came from eight different classes across four different schools); the number of children from each of the eight classes who did not take part in the study at T3 ranged from 1–5. Despite the high drop-out rate in the study, children who dropped out did not differ from the final sample in their basic characteristics (see Table S1 in Supplementary Materials).

2 Reaction time data was recorded for only three tasks (number comparison, daily events, and number ordering at T2). We did not analyse these results further, as reliability estimates for RT for young children tend to be lower than those for accuracy.

3 Due to high attrition rates at Time 3, our final sample size was slightly smaller than this.

## Supplementary Materials

The following supporting information can be downloaded at: https://www.mdpi.com/article/10.3390/jintelligence11110211/s1, https://doi.org/10.17605/OSF.IO/JFNQC. Table S1: Table demonstrating that in terms of demographics, IQ and mathematical performance, there were no significant differences at T1 between the sixty children who completed the study, and the thirty children who did not complete the study; Table S2: Pearson’s and Bayesian correlations between math anxiety and both overall math performance, and performance on the different domains of the math performance assessment at T3; Table S3: Zero-order and Bayesian correlations between MA and mathematical performance at T3, and the measures at T1 and T2; Table S4: Table showing correlations between the measures at T1, T2 and T3.
