# Supplementary material for: Basic Symbolic Number Skills, but Not Formal Mathematics Performance, Longitudinally Predict Mathematics Anxiety in the First Years of Primary School
Source: J Intell. 2023 Nov 1;11(11):211. doi: 10.3390/jintelligence11110211 (PMC10672261; doi:10.3390/jintelligence11110211)
Supplement: Supplementary file 1 [file jintelligence-11-00211-s001.zip › jintelligence-2500397-supplementary.pdf]

Supplementary Materials

Table S1. Table demonstrating that in terms of demographics, IQ and mathematical performance, there were no significant differences at T1 between the sixty children who completed the study, and the thirty children who did not complete the study.

	T3 sample (*n* = 60)	Withdrew (*n* = 30)	
	Mean *(SD)*	Mean *(SD)*	*p*
Age (in months)	59.33 *(3.82)*	59.17 *(3.61)*	.843
Gender (1=*Male*, 2=*Female*)	1.52 *(.50)*	1.40 *(.50)*	.302
Deprivation (median)	10.09 *(13.54)*	11.00 *(13.39)*	.991
Vocabulary (raw score)	15.93 *(7.03)*	16.37 *(6.31)*	.776
Block design (Raw score)	24.04 *(3.37)*	23.80 *(3.76)*	.446
Parental education[1]	4.69 *(1.44)*	4.69 *(1.32)*	.987
Maths performance	23.80 *(4.31)*	22.13 *(5.78)*	.486

Table S2. Pearson's and Bayesian correlations between math anxiety and both overall math performance, and performance on the different domains of the math performance assessment at T3.

	Math anxiety		
	r	BF_{10}	BF_{01}
Formal math performance (Raw)	-.31*	5.380	0.186
Counting and understanding number	-.35**	12.434	0.080
Knowing and using number facts	-.30*	4.418	0.226
Calculating	-.28*	3.210	0.312
Understanding shape	-.07	0.263	3.807
Measuring	-.07	0.267	3.748
Handling data	-.08	0.289	3.464

* *p* < .05, ** *p* < .01, *** *p* < .001

[1] 0 = Other, 1 = None, 2= GCSE, 3 = A-Level, 4 = Higher Education Certificate, 5 = Undergraduate degree, 6 = Postgraduate degree

Table S3. Zero-order and bayesian correlations between MA and mathematical performance at T3, and the measures at T1 and T2.

	Formal math performance (T3)			Math anxiety (T3)		
	r	BF_{10}	BF_{01}	*r*	BF_{10}	BF_{01}
OPQ (T1)	.21	1.03	0.97	.01	0.15	6.55
Order WM (T1)	.28*	3.23	0.31	.07	0.11	8.89
Order WM (T2)	.30*	4.62	0.22	.08	0.11	9.41
Daily events (T1)	.41**	62.23	0.02	-.12	0.40	2.47
Daily events (T2)	.39**	30.95	0.03	-.01	0.17	6.01
Number ordering (T1)	.25	1.73	0.58	.13	0.09	11.45
Number ordering (T2)	.49***	663.18	<0.01	-.35**	13.56	0.07
Counting (T1)	.44***	143.21	<0.01	-.24	1.67	0.60
Counting (T2)	.20	0.95	1.05	-.17	0.63	1.60
Non-symbolic addition (T1)	.23	1.39	0.72	-.22	1.26	0.79
Non-symbolic addition (T2)	.45***	197.52	<0.01	-.22	1.15	0.87
Number comparison (T1)	.14	0.49	2.05	-.37**	20.90	0.05
Number comparison (T2)	.32*	6.25	0.16	-.04	0.21	4.87
Number line estimation (T1)	-.35**	12.60	0.08	.12	0.38	2.61
Number line estimation (T2)	-.16	0.60	1.67	.07	0.26	3.83
Formal math performance (T1)	.47**	340.31	<.001	.02	0.15	6.79
Formal math performance (T2)	.58**	28939.12	<.001	-.19	0.82	1.22

* $p < .05$, ** $p < .01$, *** $p < .001$. *Task Abbreviation:* OPQ: Order-Processing Questionnaire. WM: Working memory.

Table S4. Table showing correlations between the measures at T1, T2 and T3.

	(1)	(2)	(3)	(4)	(5)	(6)	(7)	(8)	(9)	(10)	(11)	(12)	(13)	(14)	(15)	(16)	(17)	(18)	(19)	(20)	(21)
(1) Vocabulary (T1)	-																				
(2) Block design (T1)	.14	-																			
(3) OPQ (T1)	.16	.11	-																		
(4) Order WM (T1)	.23	.23	.17	-																	
(5) Order WM (T2)	-.002	.17	.24	.57***	-																
(6) Daily events (T1)	.44***	.22	-.16	.34**	.11	-															
(7) Daily events (T2)	.29*	.22	-.06	.24	.04	.46***	-														
(8) Number ordering (T1)	.24	.19	.34**	.37**	.30*	.17	.15	-													
(9) Number ordering (T2)	.41**	.09	-.05	.54***	.40**	.47***	.24	.25	-												
(10) Counting (T1)	.32*	.10	.14	.51***	.46**	.29*	.13	.30*	.54***	-											
(11) Counting (T2)	.22	.23	.18	.41**	.36**	.25	.23	.43**	.40**	.28*	-										
(12) Non-symbolic addition (T1)	.28*	.11	-.20	.11	-.002	.28*	.16	.15	.27*	.02	.33**	-									
(13) Non-symbolic addition (T2)	.35**	.36**	-.02	.12	.20	.29*	.14	.21	.35**	.21	.31*	.38**	-								
(14) Number comparison (T1)	.17	.03	.10	.14	.08	.22	.17	.12	.17	.26*	.30*	.004	-.18	-							
(15) Number comparison (T2)	.31*	.11	.07	.28*	.22	.37**	.14	.23	.48***	.32*	.19	.18	.23	-.04	-						
(16) Number line (T1)	.04	-.27*	.05	-.18	-.33*	-.25	-.19	-.09	-.22	-.29*	-.27*	-.08	-.10	-.25*	-.13	-					
(17) Number line (T2)	.13	-.08	.03	.06	.03	-.12	-.02	.07	.06	-.09	.21	.19	.04	.06	.07	.06	-				
(18) Math performance at T1	.41**	.15	.18	.23	.30*	.42***	.25	.37**	.38**	.49***	.47***	.17	.34**	.07	.45***	-.12	.20	-			
(19) Math performance at T2	.48***	.32*	.24	.10	.17	.39**	.23	.46***	.32*	.38**	.46***	.27*	.46***	.11	.42***	-.17	.06	.70***	-		
(20) Math performance at T3	.52***	.50***	.21	.28*	.30*	.41**	.39**	.24	.49***	.44**	.20	.23	.45***	.14	.32*	-.35**	-.16	.47***	.58***	-	
(21) Math anxiety (T3)	-.26*	-.20	.01	.07	.08	-.12	-.01	.13	-.35**	-.24	-.17	-.22	-.22	-.37**	-.04	.12	.07	.02	-.19	-.31*	-
